# Supplementary material for: Deficiency in class III PI3-kinase confers postnatal lethality with IBD-like features in zebrafish
Source: Nat Commun. 2018 Jul 6;9:2639. doi: 10.1038/s41467-018-05105-8 (PMC6035235; doi:10.1038/s41467-018-05105-8)
Supplement: Supplementary file 1 — Supplementary Information [file 41467_2018_5105_MOESM1_ESM.pdf]

Supplementary Information

Deficiency in class III PI3-kinase confers postnatal lethality with IBD-like features in zebrafish

Zhao et al., 2018

Supplementary Figure 1

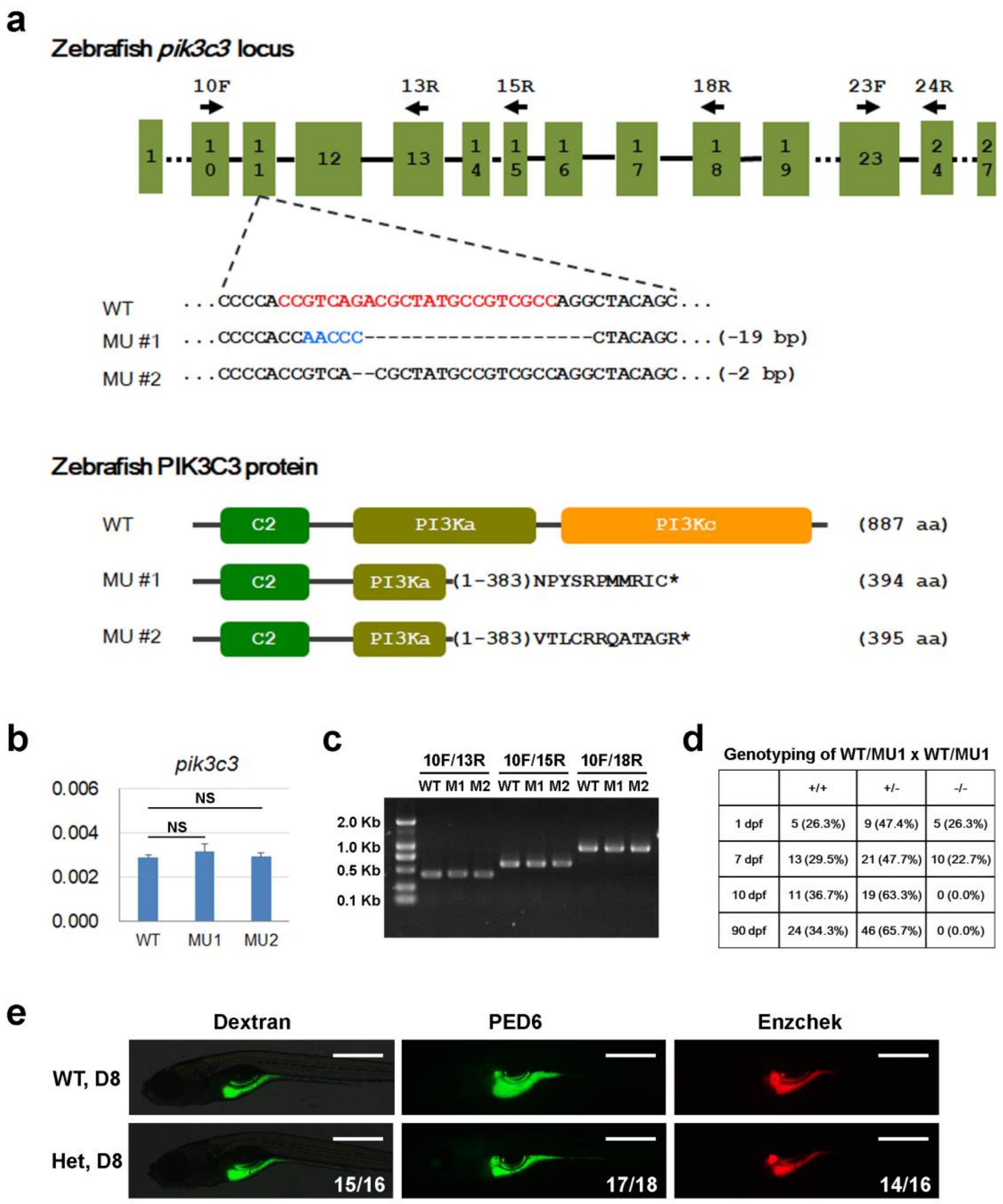

Supplementary Figure1 PIK3C3 knockout in zebrafish. (**a**) Crispr/Cas9 mediated knockout of *pik3c3*. gRNA targeting sequence in exon 11 is highlighted in red. We recovered two stably transmitted mutant alleles and they are predicted to produce truncated proteins of 394 and 395 aa respectively. (**b**) qPCR analysis with primers 23F/24R reveals that WT and mutant RNAs are expressed at similar levels. *gapdh* is the internal control and data represent mean ±SD from

three independent repeats. NS, non-significant in one-way ANOVA with Dunnett's Multiple Comparison. (**c**) RT-PCR with the indicated primer pairs generate only one band in each mutant, indicating mutant RNAs are spliced normally. (**d**) Genotyping results of WT, heterozygote and mutant at the indicated stages. (**e**) The digestive activities in heterozygous embryos are normal at 8 dpf as revealed by Dextran, PED6 and Enzchek staining. Assays were performed as described in Fig. 1b.

Supplementary Figure 2

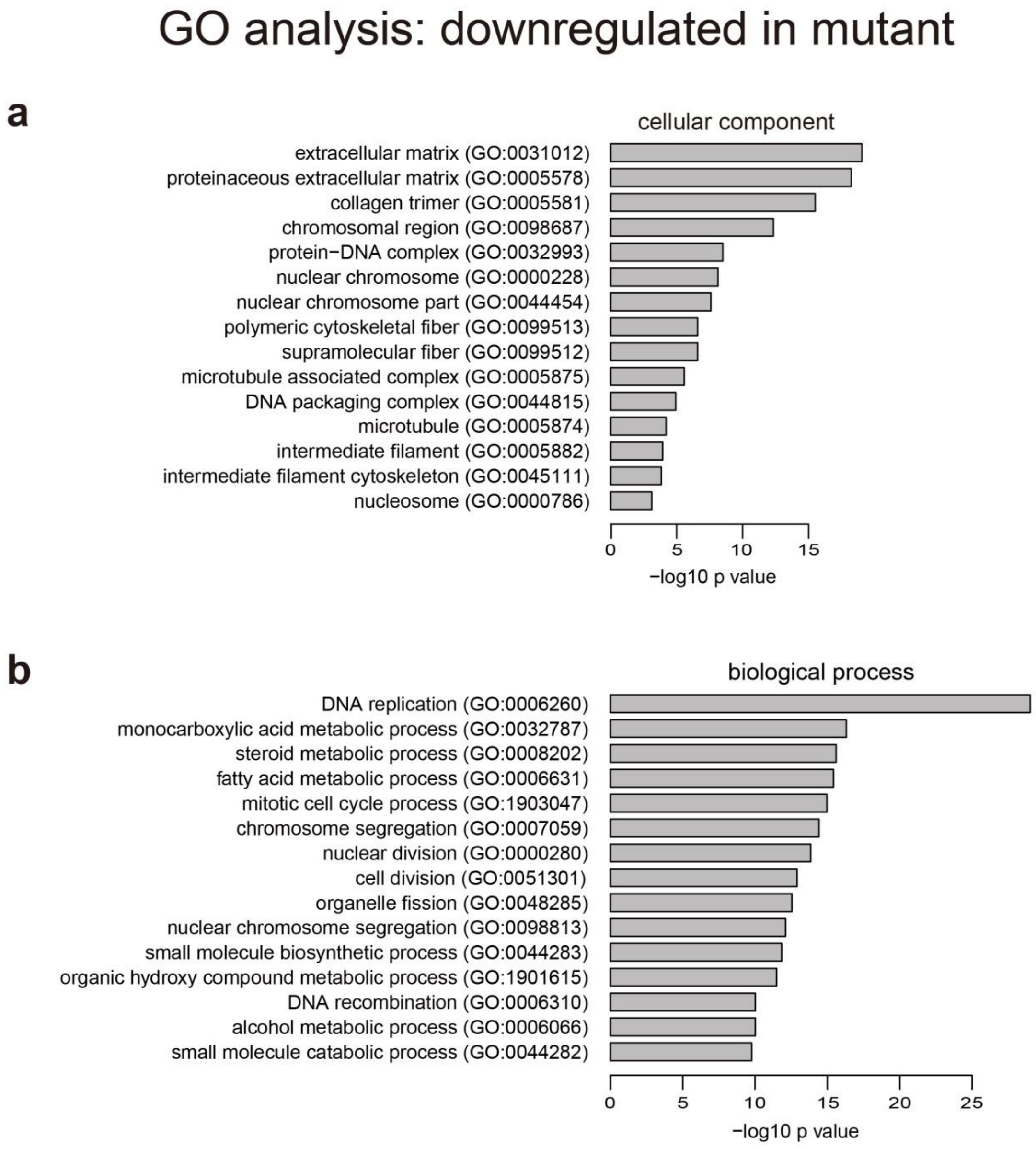

Supplementary Figure 2 Gene ontology analysis. (**a**) Cellular components down-regulated in *pik3c3* mutants at 8 dpf. Extracellular matrix related genes are dramatically suppressed in the mutants. (**b**) Biological processes down-regulated in *pik3c3* mutants at 8 dpf. DNA replication and metabolic processes associated with liver or gut are compromised in the mutants.

Supplementary Figure 3

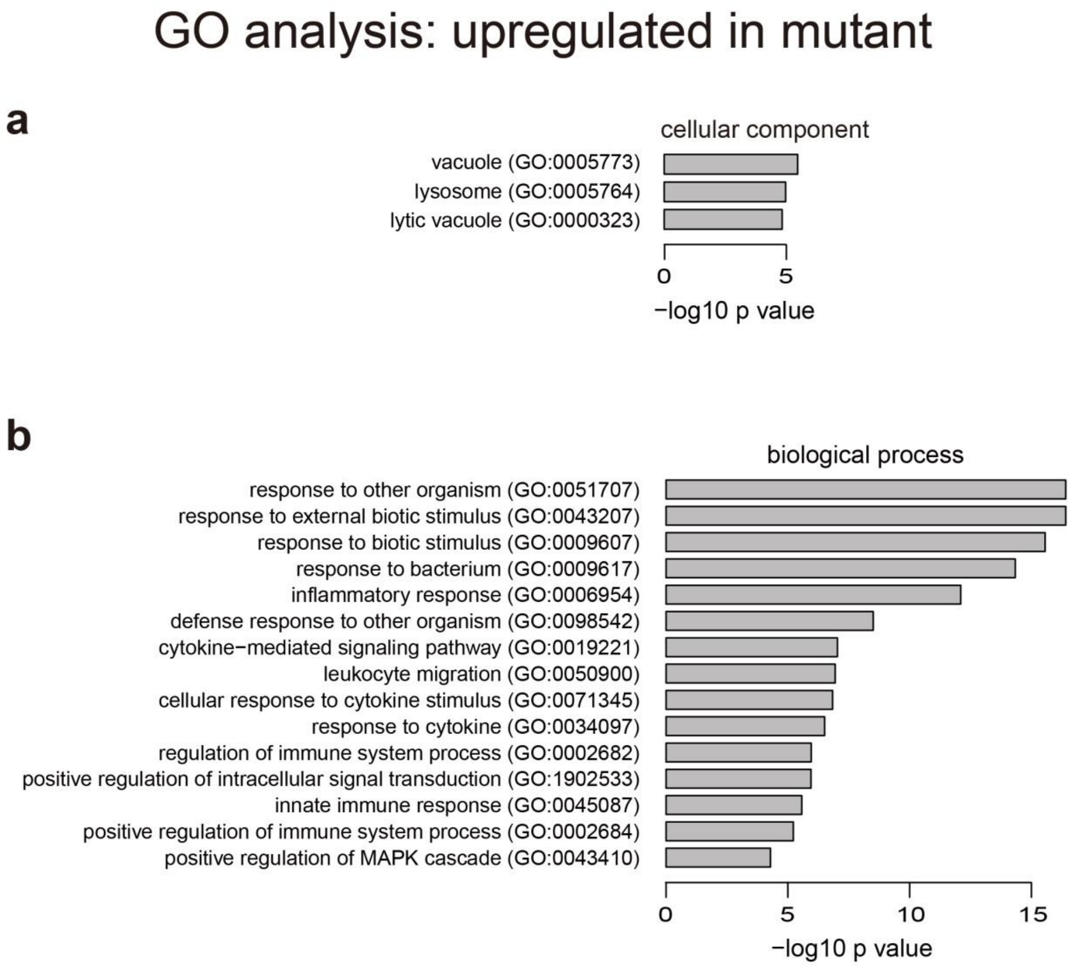

Supplementary Figure 3 Gene ontology analysis for cellular components or biological processes up-regulated in *pik3c3* mutants at 8 dpf. Inflammation and innate immune response are the major biological processes stimulated in the mutants.

Supplementary Figure 4

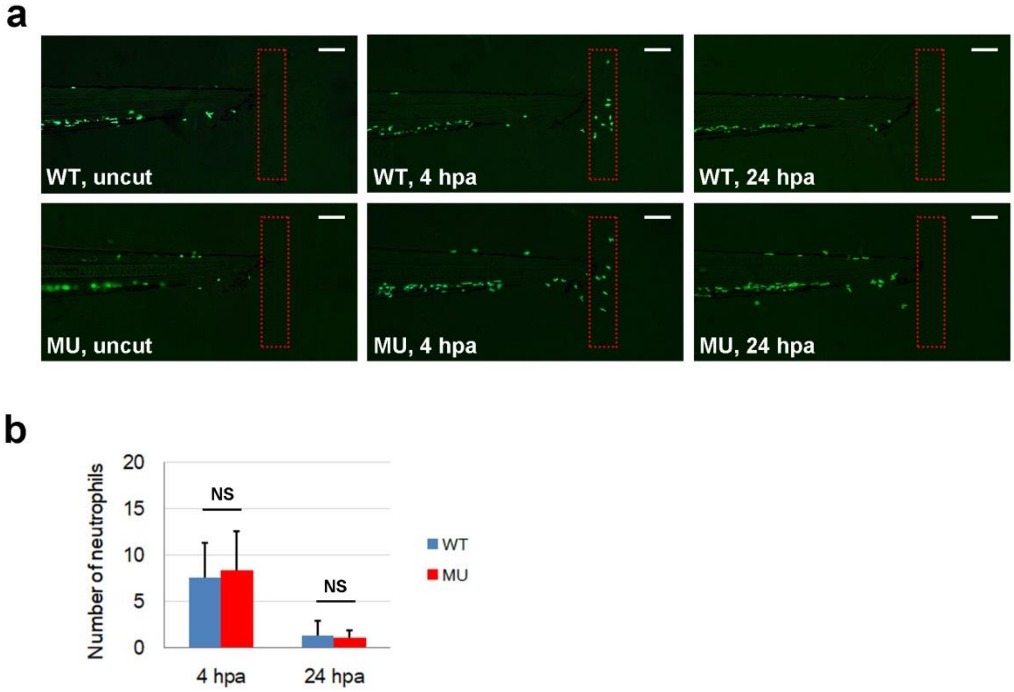

Supplementary Figure 4 Migration of mpx-GFP cells after tailfin amputation in control sibling and *pik3c3* mutant embryos. (**a**) Representative distributions of mpx-GFP cells in uncut, 4 or 24 hrs post amputation (hpa) embryos. Scale bars, 100 µm. (**b**) Average numbers of mpx-GFP cells in the outlined tail regions in (a). Data represent mean ±SD of 17 (for WT) or 13 (for MU) embryos. *p*-value calculated by one-way ANOVA with Tukey's Multiple Comparison (NS, non-significant).

Supplementary Figure 5

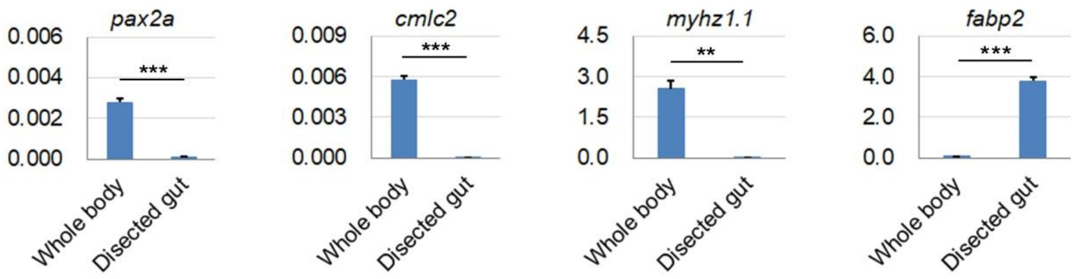

Supplementary Figure 5 qRT-PCR analysis of gene expression levels in whole embryo or dissected gut of 8 dpf. *pax2a* (*paired box 2a*) is a neuronal and pronephric duct expressed gene, *cmlc2* (*cardic myosin, light chain 7*) and *myhz1.1* (*myosin, heavy polypeptide 1.1*) are cardiac and skeletal muscle markers, *fabp2* (*fatty acid binding protein 2*) is intestinal marker. Data are normalized to *actb1* and represent mean ±SD from three independent repeats. *p*-value determined by unpaired two-tailed Student’s t-test (***p*<0.01; ****p*<0.001).

Supplementary Figure 6

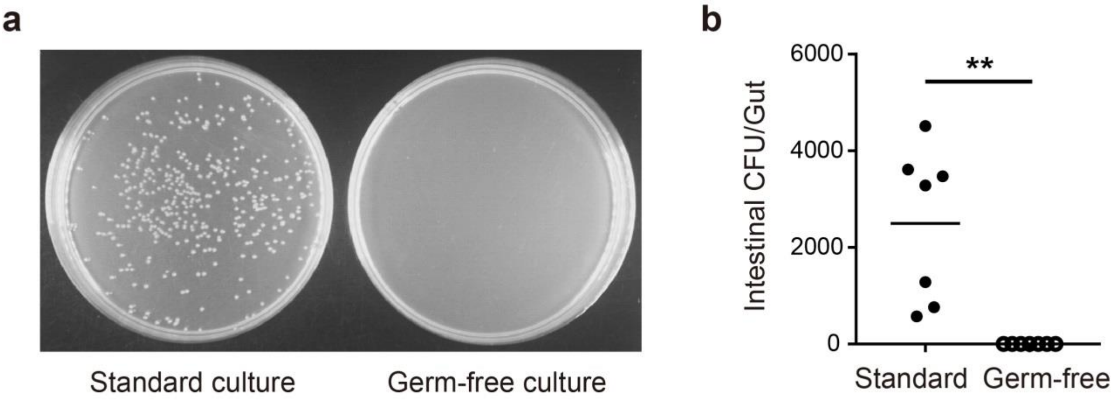

Supplementary Figure 6 Analysis of intestinal bacteria of embryos cultured under either standard or germ-free conditions. (**a**) A represent image of gut homogenate derived bacterial colony on LB agar plate. (**b**) Quantification of (a). Each dot represents the number of colony derived from one gut homogenate. The numbers of colony vary from hundreds to thousands for 8 dpf embryos under standard culture condition while no colony is detected under germ-free condition (**p<0.01 by unpaired two-tailed Student's t-test).

Supplementary Figure 7

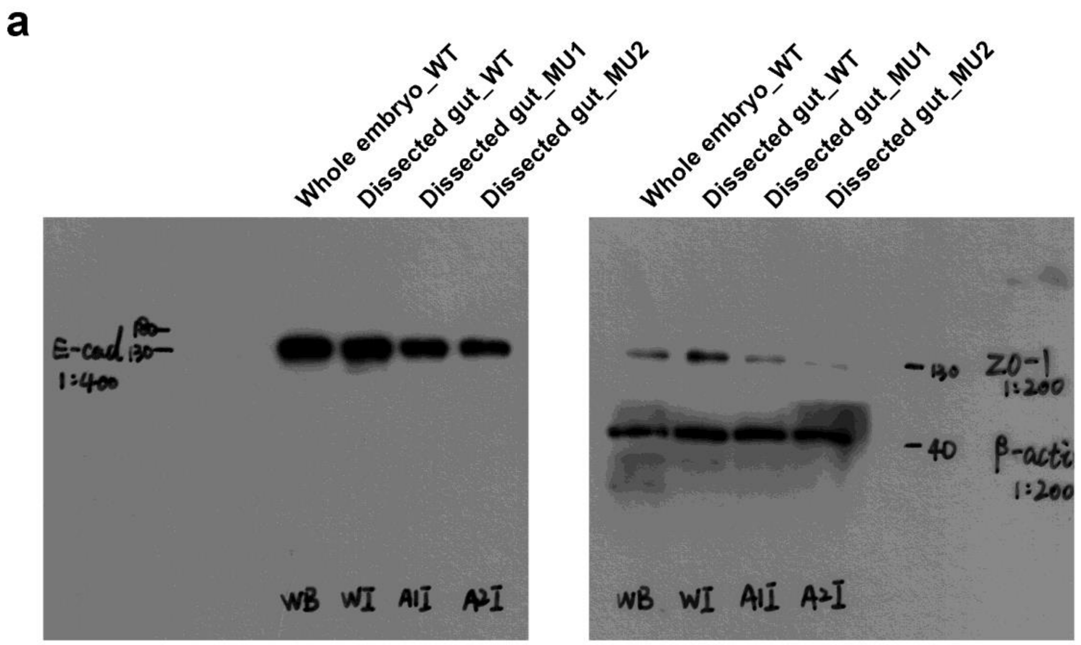

(for Figure 6b)

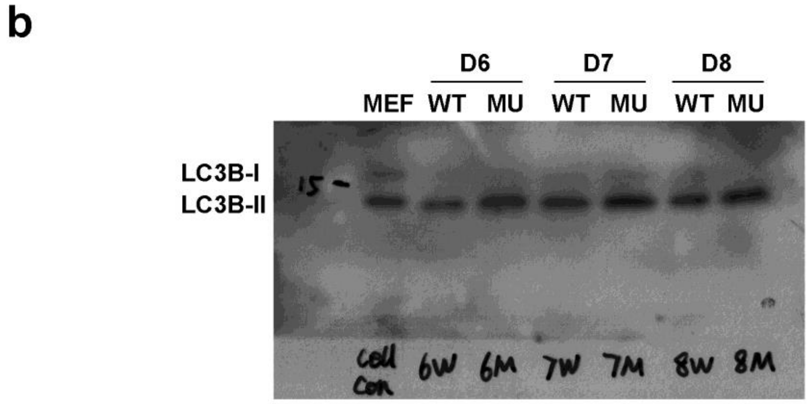

(for Figure 7a)

Supplementary Figure 7 Uncropped immunoblots used in the main figures.

Supplementary Table 1 List of PCR primers used in this study

gene	Forward(5'-3')	Reverse(5'-3')
actb1	AGATCTTCACTCCCCTTGTTCAC	ATAGGAGTCTTTCTGTCCCATGC
apoa1a	ACTCTTCTCTTGGCCTTGGG	TCCAGGTTGTCAAGGGCTTT
atg16l1	AAACGTCACCGAGCAGACAG	CGAGCTGAACGAATTCACGG
card9	GTTTGGTTATCAGACGCCGC	TCTTGCGGTACACATCTGGG
cdh1	GGCTTGTGTAACAACTGTGGG	GCCACTGTGAAGGTGATTTCG
cmlc2	TTGTTCGACCCTAATGCCACA	AAGCCTGGTCAACCTCTTCTG
cxcl8a	TGTTTTCCTGGCATTTCTGACC	TTTACAGTGTGGGCTTGGAGGG
fabp10a	AGAAGCTCAAGTGCATCGTCA	CTGGATGTGGGAGAATCGGT
fabp2	GAAGTCAGCACTTTCCGCAC	TGTGAAAGTCCCCTTGAGCG
gapdh	GTTGTGGAGTCTACTGGTGTCTT	CAGTGCTCATAAGACCTTCAACG
hnf4a	TACTAGGAGCTGCCAAACGC	TTCTTACAGCCACACGGCTC
hp	CTGATGCTACAGCCTCTACGG	GATGTGTTCTGGAAGCCTGGA
hspa5	TCTGTGCAGCAGGACATCAAA	TTGGTCAAAACCATGGCGGA
igfbp1a	TTGAAGAGAGGTGACCCGTG	TTGGCTGTGGTTAGGCTCG
igfbp1b	CACCTGCTGAGCCTGAACAG	GAGAAGCTCAGTGTGACACGG
il1b	GGCTGTGTGTTTGGGAATCT	TGATAAACCAACCGGGACA
ins	CCCAAGAGAGACGTTGAGCC	CAGCCACCTCAGTTTCCTGG
irgm	TATCACCAAGGTGCTCGCTG	AGCAGTGGAGACAACGAACT
lect2l	TGTGTGTACTGATGGAGCCAC	CCGGACAAACGATCTGGCTT
mmp9	GCTCAACCACCGCAGACTAT	GTGCTTCATTGCTGTTCCCG
mpx	CTACATGGCACAAACGCTGAG	CTCGTCTTGAGTGAGCAGGTT
myhz1.1	GATGCTGTTAAAGGCGTCCG	CAGATCCTGCAGCCTGTTGA
nod2	GCAAGGAGGGGGTTGATTGT	TCTGCATTCTTGCTGGCTCA
pax2a	CGTTTGTGACAACGACACAGT	GCTGTGGAAAGAGGTGTTCCT
slc15a1b	TTGGTTCCCCATGGCAAAGT	TCCAAAGCCCAGCTGCATAA
tnfa	CAGGGCAATCAACAAGATGG	TGGTCCTGGTCATCTCTCCA
try	CGCCCAAATCAACAGCTACG	AATGGGAGCATTCAGGCACA
ttc7a	GAGACTGCTGTGTCTCGTCTG	ATGAACAACTCCCCTGCCTG
vil1	ACGCAGACTTCTGCATGTGA	AGCAGGAACACATCGCCTTT
zo-1	GCTTACCTCACTGTGCGTCT	AGGTAGTTGGGATCTCCGGG
23F	GCATGCAGAGCGAGCAATAC	
24R		TCCAGCGCAATGTCTGGAAT
10F	ACAGCTGAGCTCTGAGGAAC	
13R		CTGGGCATCACTGCAATAGC
15R		CACATTGAGGTACATGTCATGGG
18R		CCCCATGTTTGAAGATGACAGG
hUVRAG	TGACAATTCGTTGCAGGCAG	AGGCAACTTGACACCGCATA
hATG14	GCGTCTGGCAAATCTTCGAC	TCTGAAGACACATCTGCGGG
hPIK3C3	GCTGTGCTGGATATTGCGTG	AAGAGGCTTTGGATCCCGAC
hACTB	CCCAGAGCAAGAGAGG	GTCCAGACGCAGGATG

Supplementary Table 2 List of antibodies used in this study

Antibody	Host	Clone	Source	Catalog No.	Dilution for IF	Dilution for WB
ATP1A1	Rabbit		Cell Signaling	#3010	1:50(z)	
beta-Actin	Mouse	AC-15	Sigma	A5441		1:1000(h)<br>1:400(z)
E-Cadherin	Mouse		BD Biosciences	610182	1:100(h)<br>1:50(z)	1:1000(h)<br>1:200(z)
HSPA5	Rabbit	C50B12	Cell Signaling	#3177	1:50(z)	
LAMP1	Rabbit		Abcam	ab24170	1:50(z)	
LC3B	Rabbit		Abcam	ab51520		1:1500(z)
ZO-1	Mouse	1A12	Thermo Fisher	33-9100	1:100(h)<br>1:50(z)	1:400(z)

Note: (z): zebrafish samples; (h): Caco2 samples.
